# Successful downstream application of the Paxgene Blood RNA system from small blood samples in paediatric patients for quantitative PCR analysis

**DOI:** 10.1186/1471-2172-8-20

**Published:** 2007-09-12

**Authors:** Enitan D Carrol, Fiona Salway, Stuart D Pepper, Emma Saunders, Limangeni A Mankhambo, William E Ollier, C Anthony Hart, Phillip Day

**Affiliations:** 1 Malawi-Liverpool-Wellcome Trust Clinical Research Programme, PO Box 30096, Blantyre, Malawi, Africa; 2 Centre for Integrated Genomic Medical Research, (CIGMR), Stopford Building, The University of Manchester, Oxford Road, Manchester, M13 9PT, UK; 3 Paterson Institute for Cancer Research, The University of Manchester, Wilmslow Rd, Manchester, M20 4BX, UK; 4 Department of Paediatrics, College of Medicine, University of Malawi, Africa; 5 Division of Child Health, University of Liverpool, Royal Liverpool Children's NHS Trust, Alder Hey, Eaton Road, Liverpool L12 2AP, UK; 6 Division of Medical Microbiology, The University of Liverpool, Duncan Building, Daulby Street, Liverpool L69 3GA, UK; 7 Institute for Analytical Sciences Bunsen-Kirchoff-Str. 11, 44139, Dortmund, Germany

## Abstract

**Background:**

The challenge of gene expression studies is to reliably quantify levels of transcripts, but this is hindered by a number of factors including sample availability, handling and storage. The PAXgene™ Blood RNA System includes a stabilizing additive in a plastic evacuated tube, but requires 2.5 mL blood, which makes routine implementation impractical for paediatric use.

The aim of this study was to modify the PAXgene™ Blood RNA System kit protocol for application to small, sick chidren, without compromising RNA integrity, and subsequently to perform quantitative analysis of ICAM and interleukin-6 gene expression.

Aliquots of 0.86 mL PAXgene™ reagent were put into microtubes and 0.3 mL whole blood added to maintain the same recommended proportions as in the PAXgene™ evacuated tube system. RNA quality was assessed using the Agilent BioAnalyser 2100 and an in-house TaqMan™ assay which measures GAPDH transcript integrity by determining 3' to 5' ratios. qPCR analysis was performed on an additional panel of 7 housekeeping genes. Three reference genes (HPRT1, YWHAZ and GAPDH) were identified using the GeNORM algorithm, which were subsequently used to normalising target gene expression levels. ICAM-1 and IL-6 gene expression were measured in 87 Malawian children with invasive pneumococcal disease.

**Results:**

Total RNA yield was between 1,114 and 2,950 ng and the BioAnalyser 2100 demonstrated discernible 18s and 28s bands. The cycle threshold values obtained for the seven housekeeping genes were between 15 and 30 and showed good consistency. Median relative ICAM and IL-6 gene expression were significantly reduced in non-survivors compared to survivors (ICAM: 3.56 vs 4.41, p = 0.04, and IL-6: 2.16 vs 6.73, p = 0.02).

**Conclusion:**

We have successfully modified the PAXgene™ blood collection system for use in small children and demonstrated preservation of RNA integrity and successful quantitative real-time PCR analysis.

## Background

The main challenge of quantitative gene expression studies is to extract sufficient usable messenger ribonucleic acid (mRNA), to avoid degradation and permit analysis for calculation of exact numbers of transcript. The processes of sample collection, transport, processing and storage may result in significant degradation of mRNA [1]. Because of the lability of mRNA in clinical samples, it is essential that the integrity of the mRNA is assessed before proceeding with downstream applications such as reverse transcription real-time quantitative polymerase chain reaction (RTqPCR) and micro-array analyses. Both techniques are highly sensitive and rely on meticulous and consistent sample processing [2,3]. The correct interpretation of transcript abundance requires stabilisation of the transcriptome at the point of sample collection, through storage and transport, in order for gene expression to be detected in a reproducible manner [4].

The PAXgene™ Blood RNA System (PreAnalytiX, QIAGEN, Germany), includes a stabilizing additive in an evacuated blood collection tube called the PAXgene™ Blood RNA Tube, and also sample processing reagents in the PAXgene™ Blood RNA Kit. The additive in the PAXgene™ tube reduces RNA degradation of 2.5 mL of blood in the evacuated tube, and furthermore, the RNA in whole blood has been shown to be stable at room temperature for 5 days, following storage for up to 12 months at -20°C and -80°C, and also after repeated freeze-thaw cycles [5].

Recent studies have shown that RNA can be detected and quantified in peripheral blood collected into PAXgene™ collection tubes, and has allowed comparison of RNA levels between patients with diabetic retinopathy [6], thyroid cancer [7] and healthy controls.

The PAXgene™ tube reagents have been used to assess inflammatory responses in vivo using 2.5 mL of whole blood and in vitro using 200 μL of heparinised blood [3]. An assay using branched DNA has been described to quantitatively measure mRNA expression from small blood volumes, but this method did not utilise the PAXgene™ Blood RNA system [8].

Children with pneumococcal disease in developing countries such as Malawi often present late, and are critically ill by the time they reach hospital. It is difficult to collect 2.5 mls of blood from such children in the evacuated PAXgene™ Blood RNA Tube using the suggested protocol, and therefore we sought to optimise the protocol for use in small, sick children by employing smaller blood volumes in paediatric microtubes, and to subsequently use the RNA for the quantification of cytokine and mediator responses to invasive pneumococcal disease.

Intracellular adhesion molecules (ICAMs), vascular cell adhesion molecules (VCAMs), β_2 _integrins (CD11a/CD18 and CD11b/CD18), P-selectin and E-selectin are involved in the adhesion of circulating leucocytes to endothelial cells. P-selectin and ICAM-1 (but not VCAM-1) are up-regulated in experimental pneumococcal meningitis [9]. Pneumolysin, a major virulence factor for *Streptococcus pneumoniae *increases ICAM-1 mRNA [10]. Fucoidin, an L-selectin blocker profoundly reduces leucocyte rolling (a precondition for leucocyte adhesion to vascular endothelium), pleocytosis and increased CSF protein levels in experimental pneumococcal meningitis, thereby reducing leucocyte dependent damage in bacterial meningitis [11]. Interleukin-6 (IL-6) production is a marker of sepsis-related mortality and poor outcome in models of pneumococcal disease [12] and increased IL-6 production has been shown in the lungs of pneumococcal-infected mice [13]. On the basis of existing knowledge of the biological function of the ICAM and IL-6 genes in the host response to pneumococcal infection, we conducted a pilot study to quantify ICAM and IL-6 gene expression in Malawian children presenting with invasive pneumococcal disease. Before proceeding with RT qPCR analysis, we validated the scaled-down method by assessing mRNA integrity using an in-house assay that measures three regions of the GAPDH transcript.

## Results

### Whole blood extraction from healthy volunteers

**Figure 1:**
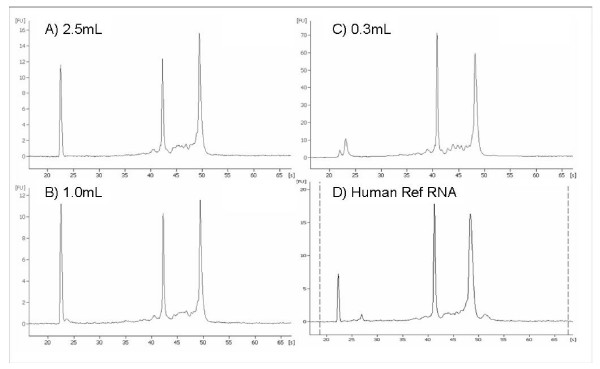
Agilent BioAnalyser 2100 traces of total RNA samples. Different volumes of peripheral blood were processed using PAXgene reagent as described in the text. A) Full scale 2.5 mL peripheral blood extraction B) 1.0 mL peripheral blood scaled down extraction C) 0.3 mL peripheral blood scaled down extraction D) Stratagene Universal RNA. The 2.5 mL and 1.0 mL extractions were run on eukaryote total RNA Nano chips and the 0.3 mL extractions and the Universal RNA shown were run on Pico chips. The 18s and 28s RNA peaks can be seen at approximately 42 and 48 seconds respectively.

Whole venous blood was collected from healthy volunteers and either 2.5, 1.0 or 0.3 mL added to the Paxgene blood RNA reagent in the same ratio as the manufacturers guidelines. The total RNA yield from 2.5, 1.0 and 0.3 mL of whole blood was 4.5 – 11.6 μg, 5.1 – 8.3 μg and 1.6 to 5.0 μg respectively. With optical density ratios (260/280) of 1.97–2.14. Figure 1 shows Agilent 2100 Bioanalyser traces for the total RNA extracted from the different volumes of whole blood. Panel A shows the full scale (2.5 mL) blood RNA extraction, panels B and C show total RNA from the low volume extractions, for comparison panel D shows the trace for Stratagene Universal RNA. The 18s and 28s RNA peaks can be seen at approximately 42 and 49 seconds respectively. Both the 2.5 mL and 1.0 mL extractions were loaded on to Eukaryote total RNA Nano chips whereas the 0.3 mL extractions were loaded on to Eukaryote total RNA Pico chips. The Bioanalyser software generates RNA Integrity Numbers (RIN) for each sample giving an estimate of the RNA integrity. Total RNA extracted from 0.3 – 2.5 mL had a RIN of between 8.2 and 9.6. RNA was reverse transcribed and assayed for a panel of reference genes to ensure expression profiles were maintained across all volumes extracted.

### Whole blood extraction from patients with invasive pneumococcal disease and controls

Whole venous blood samples were collected from 87 children with confirmed invasive pneumococcal disease. A total of 48 children (55%) were male, and the age range was 0.17 to 13 years, median age 3.25 years. There were 25 (28.7%) non-survivors. Of the children with IPD, 75 had meningitis(86%) and 12 had pneumonia (14%).

**Figure 2:**
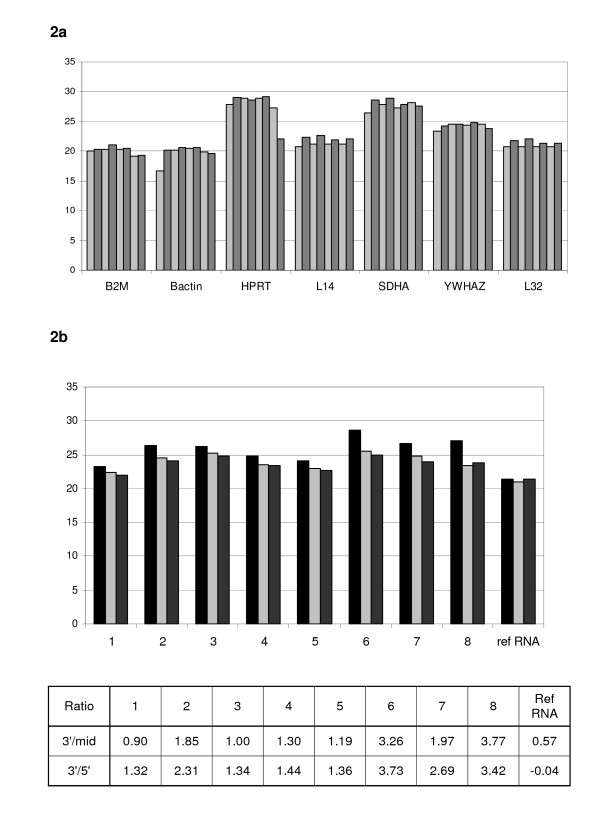
Real time qPCR results obtained using small volumes of whole blood. Figure 2a shows the Ct values obtained when 8 RNA extracts were assayed for 7 housekeeper genes. Figure 2b shows the Ct values for 3 assays detecting GAPDH at 3' (▯), mid (▯) and 5' (▯) positions.

Total RNA yield from 0.3 mL of whole blood varied between 1.1 and 2.9 μg with 260/280 ratios of 1.91 – 2.03. Eight small volume blood samples were processed using the method described below and amplified for the 3 GAPDH assays. Figure 2a shows that in all cases a good signal is detected across the whole GAPDH gene, thereby indicating that full length mRNA has been isolated. Figure 2b shows the raw Ct values obtained for the 8 samples with a panel of 7 additional housekeeper genes. The samples show excellent consistency in the expression levels for all the housekeeper assays further indicating that all the RNA samples have good mRNA integrity. The RT qPCR assays were shown to be a more sensitive method of RNA QC analysis for samples and could be used in place of the Bioanalyser.

**Figure 3:**
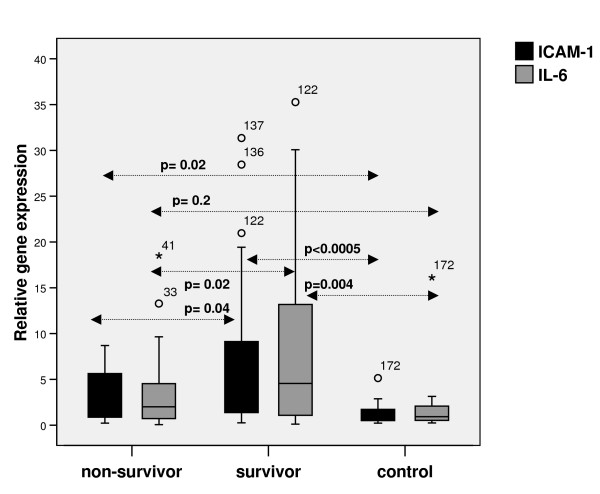
Box and whisker plot of relative gene expression in the ICAM-1 and IL-6 genes in survivors (n = 62), non-survivors (n = 25) and controls (n = 16). The dark line represents the median, and the box represents the interquartile range. The whiskers represent the range, and outliers are depicted as small circles.

The RNA yields were low but sufficient cDNA was produced to perform RT qPCR experiments to quantify ICAM and IL-6 gene expression. Relative gene expression (2^-ΔΔCt^) was significantly higher in survivors compared to non-survivors and controls (ICAM: p < 0.0005 and IL-6: p = 0.003, Kruskal Wallis). Relative gene expression was significantly lower than in non-survivors than survivors; ICAM: median (IQR); 3.56 (0.82 – 5.72) versus 4.41 (1.44 – 9.57), and IL-6: median (IQR); 2.16 (0.71 – 5.72) versus 6.73 (1.17 – 14.93). Relative gene expression was significantly lower in controls than cases ICAM: 1.00 (0.54 – 1.79) versus 4.17(1.29 – 8.06) and IL-6: 0.92 (0.49 – 2.28) versus 4.32(0.81 – 13.27) (p < 0.0005 and p = 0.01 respectively) (Figure 3).

## Discussion

In this study using 0.3 mL of whole blood we have modified the PAXgene™ Blood RNA System for use in small sick children. Our results show that in all cases a near equivalent signal is detected across the tested regions of the GAPDH gene, thereby indicating that full length mRNA has been isolated. In turn, this result in combination with the similar levels of expression seen for 7 reference genes suggests that other transcripts are useable for RT qPCR analyses. We believe that this approach to assessing transcript integrity will be of general utility in transcriptional analysis. The RNA was then successfully applied for downstream quantitative gene expression of the ICAM-1 and IL-6 genes, using RT qPCR.

Our data show that ICAM-1 and IL-6 expression are increased in cases compared to healthy controls, and that expression is also increased in survivors compared to non-survivors with invasive pneumococcal disease. This is consistent with a study by Rieckmann et al. where elevated soluble ICAM-1 (sICAM-1) levels were seen in the cerebrospinal fluid of patients with bacterial meningitis [14]. Increased IL-6 expression has been shown in a rat model of pneumocococal infection [15], and IL-6 GG homozygous patients (with increased IL-6 transcription and production) were less likely to develop extrapulmonary pneumococcal infection (as marker of impaired clearance of bacteria) [16]. ICAM-1 plays an important role in tight binding of leucocytes to the endothelium, and IL-6 is required for the clearance of bacteria. Our data would suggest that under-expression of both these mediators may be detrimental to the host.

The PAXgene™ Blood RNA System allows stabilisation of the transcriptome at the point of collection, at the bedside, and thus facilitates the ready access of gene expression studies to the clinical research scientist. The samples do not need to be processed immediately, and can be stored at room temperature for up to 24 hours before freezing or extraction, although different handling conditions have been shown to contribute minimally (0.09%) to differences in gene expression levels [4].

Previous researchers have found that the PAXgene™ Blood RNA System produced reliable gene expression profiles using the Affymetrix GeneChip^® ^system, and showed small but significant differences in gene expression between two sample handling methods. Samples which were freshly extracted had higher DNA contamination, and lower total RNA yield than those which were frozen at -20°C before extraction, but the authors do not explain this finding [4]. In this study we have chosen a consistent method of sample processing (incubate at room temperature for a maximum of two hours and freeze at -80°C before extraction) to reduce variability. Another study showed that a longer incubation time of 24 hours in PAXgene™ tubes before extraction produced a higher total RNA yield than incubation times of 4 hours. This might be another useful strategy for application to small volume samples such as those drawn from children [17].

The method described in this study produced sufficient mRNA for successful downstream quantitative gene expression analysis, but did not consistently produce sufficient RNA for application on a micro-array using Affymetrix GeneChips^®^.

## Conclusion

In summary the results presented here show a robust efficient system for the collection and processing of blood samples to allow accurate expression profiling. We have demonstrated that 0.3 mL of whole blood can be routinely used as the basis for transcriptional profiling studies thereby making this approach available to neonatal and paediatric studies, but may have other clinical applications where availability of blood is limited, such as small animal veterinary science.

## Methods

### Patient recruitment

Children in this study were admitted to the Queen Elizabeth Central Hospital, Blantyre, Malawi, and recruited into a prospective observational study of host determinants of invasive pneumococcal disease susceptibility and severity in Malawian children, the details of which have been described elsewhere [18]. Controls were taken from healthy, afebrile children from the same villages as the cases. The study protocol was approved by the College of Medicine Research Ethics Committees, both in Blantyre, Malawi and the Local Research Ethics Committee at the Liverpool School of Tropical Medicine, United Kingdom. Written informed consent was obtained from parents or guardians before children were included into the study.

### Sample collection

Peripheral whole blood (0.3 mL) from venepuncture was dispensed into micro-tubes pre-aliquoted with PAXgene™ reagent (0.86 mL), keeping the blood:reagent ratio the same as in the PAXgene™ Blood RNA Tubes [Qiagen]. The sample was gently inverted and stored at -80°C within two hours of collection.

### RNA extraction

RNA was extracted from whole blood using the PAXgene™ Blood RNA System Kit employing an amended version of the manufacturer's guidelines. Briefly, the samples were removed from -80°C and incubated at room temperature for 2 hours to ensure complete lysis. Following lysis the tubes were centrifuged for 10 min at 5,000 × g (Boeco M-24 centrifuge), the supernatant decanted and 500 μL of RNase-free water added to the pellet. The tube was vortexed to thoroughly re-suspend the pellet, centrifuged for 10 min at 5000 × g and the entire supernatant discarded. The remaining pelleted lysate was re-suspended in 360 μL of buffer BR1 by vortexing and the manufacturer's protocol was followed from this step.

### RNA concentration and analysis

Freshly extracted RNA was measured using a NanoDrop ND-1000 UV-visible spectrophotometer [Labtech International, Ringmer, UK]. The software displays the concentration in ng/μL. There is also a quality output, which provides 260/280 and 260/230 ratios enabling purity estimations. RNA integrity was additionally assessed using the Agilent 2100 Bioanalyser [Agilent Technologies]. Samples were loaded on to either the Eukaryote total RNA nano chip or the Eukaryote total RNA pico chip.

### Assessment of RNA integrity by qPCR

To evaluate the utility of the RNA achieved by this method we then carried out RT qPCR analysis on a panel of 10 assays on a total of 8 housekeeper genes as detailed in Table 1. As a further quality control (QC) measure with enhanced sensitivity, we developed assays to 3 regions of GAPDH; termed 3', mid and 5'. By using oligo dT for the priming step during reverse transcription it is possible to use the presence of the mid and 5' assays for GAPDH to confirm that full length transcripts are present. RNA (1 μg) was reverse transcribed using SuperScript II™ reverse transcriptase (Invitrogen Ltd, Paisley, UK) following the manufacturer's guidelines, with oligo dT_12–18 _used for priming. RT qPCR assays were designed using locked nucleic acid analogues (LNA) of fluorescence resonance energy transfer hydrolysis probes [19] employed in the Human Universal Probe Library system (Roche, Switzerland), with amplification primers obtained from MWG (Edersberg, Germany). Experiments were performed on an ABI 7900 Real-Time Sequence Detection System in 384 well format, using an EpMotion 5070 robot (Eppendorf, Germany) for plate set up.

**Table 1:** Real time PCR primers and probes

**Gene name**	**Accession No**.	**Universal Probe No**.	**Primer**	**Primer sequence**
B2M	NM_004048.2	42	sense	ttctggcctggaggctatc
			non-sense	tcaggaaatttgactttccattc
Beta Actin	NM_001101.2	11	sense	attggcaatgagcggttc
			non-sense	ggatgccacaggactccat
HPRT	NM_000194.	73	sense	tgaccttgatttattttgcatacc
			non-sense	cgagcaagacgttcagtcct
L14	BC000606	8	sense	tcctcaagtttccgcacagt
			non-sense	ggctgcccattttgtattga
L32	NM_000994.3	17	sense	gaagttcctggtccacaacg
			non-sense	gcgatctcggcacagtaag
SDHA	NM_004168.1	69	sense	agaagccctttgaggagca
			non-sense	cgattacgggtctatattccaga
YWHAZ	NM_003406.2	9	sense	cgttacttggctgaggttgc
			non-sense	tgcttgttgtgactgatcgac
GAPDH 3'	M33197_3	45	sense	acacccactcctccaccttt
			non-sense	tgacaaagtggtcgttgagg
GAPDH mid	M33197_M	45	sense	gggaaactgtggcgtgat
			non-sense	gatgaccttgcccacagc
GAPDH 5'	M33197_5	9	sense	ggaagcttgtcatcaatggaa
			non-sense	ttgattttggagggatctcg
ICAM1	NM_000201	10	sense	agcttctcctgctctgcaac
			non-sense	aatccctctcgtccagtcg
IL-6	NM_000600	40	sense	gatgagtacaaaagtcctgatcca
			non-sense	ctgcagccactggttctgt

### Reverse transcription

RNA was reverse transcribed using SuperScript II™ RNase H reverse transcriptase (Invitrogen Ltd, Paisley, UK) following the manufacturer's guidelines. Reactions were primed using 1 μL oligo (dT)_12–18 _(500 μg/mL) and took place in the presence of RNaseOUT™ (all reagents were from Invitrogen Ltd, Paisley, UK)

### Real-time quantitative PCR measurement of ICAM and IL-6 genes

The Human Universal Probe Library system [19] (Roche, Switzerland) employing proprietary LNA analogues was used for RT qPCR to measure expression levels in genes of interest. Using the Roche Online Assay Design Centre, specific primers and an associated probe were selected for the ICAM transcript. To compensate for variations in cell number, RNA isolation, reverse transcription and PCR amplification efficiency, three endogenous 'house-keeping' transcripts were chosen using the GeNORM algorithm [20]. Briefly, the cases and controls were screened for 8 reference genes, the algorithm works out the stability of each transcript, and sequentially removes the least stable transcript until the three most stable transcripts remain. These were; HPRT1 (Hypoxanthine phosphoribosyl transferase 1), YWHAZ (tyrosine 3 monooxygenase) and GAPDH glyceraldehyde 3 phosphate dehydrogenase.

cDNA was diluted 1 in 50. Each reaction comprised of 5 μL diluted cDNA, 10 μL 2× qPCR Master Mix with UNG (Eurogentec Ltd., Southampton, UK), 0.2 μL each primer (20 μM) (Metabion, Planegg-Martinsried, Germany), 0.2 μL Probe (10 μM) Human Universal Probe Library system [19] (Roche, Switzerland), and 4.4 μL of water. Samples were then amplified on an ABI 7700 PRISM SDS, and the temperature programme resembled 50°C for 2 min, 95°C for 10 min followed by 40 cycles of 95°C for 15 sec and 60°C for 1 min. The amounts of target genes expressed in a sample are normalized to the average of the three endogenous controls. This is given by ΔC_T_, where ΔC_T _is determined by subtracting the average endogenous gene C_T _value from the average target gene C_T _value. [C_T _target gene - C_T _average (endogenous gene)]. The calculation of ΔΔC_T _involves subtraction of ΔC_T _value for the controls from the ΔC_T _value for the cases [ΔC_T _target gene_(case) _- ΔC_T _target gene_(control)_]. 2^-ΔΔCt ^is the relative expression of the target gene in cases compared to controls.

### Statistics

For comparisons (between survivors and non-survivors with invasive pneumococcal disease, and healthy afebrile controls), the Mann Whitney test was used to compare two groups and Kruskal Wallis to compare three groups. Values of p < 0.005 were considered statistically significant. SPSS statistical software version 14 (SPSS, Chicago, IL) was used for all statistical analyses.

## Authors' contributions

EDC participated in the design and organisation of the study, the collection of clinical and laboratory data, laboratory work, statistical analysis and in drafting and revising the manuscript. FS participated in the design and organisation of the study, laboratory work, and in drafting and revision of the manuscript. SDP and ES participated in the laboratory work, and revision of the manuscript. LAM participated in the collection of clinical and laboratory data, and in drafting and revising the manuscript. WO and CAH participated in the design and organisation of the study, and revision of the manuscript. PD participated in the design and organisation of the study, statistical analysis and in drafting and revision of the manuscript.

All authors read and approved the final manuscript.

## References

[B1] Hartel C, Bein G, Muller-Steinhardt M, Kluter H (2001). Ex vivo induction of cytokine mRNA expression in human blood samples. J Immunol Methods.

[B2] Lockhart DJ, Winzeler EA (2000). Genomics, gene expression and DNA arrays. Nature.

[B3] Stordeur P, Zhou L, Byl B, Brohet F, Burny W, de Groote D, van der Poll T, Goldman M (2003). Immune monitoring in whole blood using real-time PCR. J Immunol Methods.

[B4] Thach DC, Lin B, Walter E, Kruzelock R, Rowley RK, Tibbetts C, Stenger DA (2003). Assessment of two methods for handling blood in collection tubes with RNA stabilizing agent for surveillance of gene expression profiles with high density microarrays. J Immunol Methods.

[B5] Rainen L, Oelmueller U, Jurgensen S, Wyrich R, Ballas C, Schram J, Herdman C, Bankaitis-Davis D, Nicholls N, Trollinger D, Tryon V (2002). Stabilization of mRNA expression in whole blood samples. Clin Chem.

[B6] Hamaoui K, Butt A, Powrie J, Swaminathan R (2004). Real-time quantitative PCR measurement of circulatory rhodopsin mRNA in healthy subjects and patients with diabetic retinopathy. Ann N Y Acad Sci.

[B7] Li D, Butt A, Clarke S, Swaminathana R (2004). Real-time quantitative PCR measurement of thyroglobulin mRNA in peripheral blood of thyroid cancer patients and healthy subjects. Ann N Y Acad Sci.

[B8] Zheng Z, Luo Y, McMaster GK (2006). Sensitive and quantitative measurement of gene expression directly from a small amount of whole blood. Clin Chem.

[B9] Winkler F, Koedel U, Kastenbauer S, Pfister HW (2001). Differential expression of nitric oxide synthases in bacterial meningitis: role of the inducible isoform for blood-brain barrier breakdown. J Infect Dis.

[B10] Thornton J, McDaniel LS (2005). THP-1 monocytes up-regulate intercellular adhesion molecule 1 in response to pneumolysin from Streptococcus pneumoniae. Infect Immun.

[B11] Granert C, Raud J, Xie X, Lindquist L, Lindbom L (1994). Inhibition of leukocyte rolling with polysaccharide fucoidin prevents pleocytosis in experimental meningitis in the rabbit. J Clin Invest.

[B12] Clatworthy MR, Smith KG (2004). FcgammaRIIb balances efficient pathogen clearance and the cytokine-mediated consequences of sepsis. J Exp Med.

[B13] Marriott HM, Hellewell PG, Cross SS, Ince PG, Whyte MK, Dockrell DH (2006). Decreased alveolar macrophage apoptosis is associated with increased pulmonary inflammation in a murine model of pneumococcal pneumonia. J Immunol.

[B14] Rieckmann P, Nunke K, Burchhardt M, Albrecht M, Wiltfang J, Ulrich M, Felgenhauer K (1993). Soluble intercellular adhesion molecule-1 in cerebrospinal fluid: an indicator for the inflammatory impairment of the blood-cerebrospinal fluid barrier. J Neuroimmunol.

[B15] Long JP, Tong HH, Shannon PA, DeMaria TF (2003). Differential expression of cytokine genes and inducible nitric oxide synthase induced by opacity phenotype variants of Streptococcus pneumoniae during acute otitis media in the rat. Infect Immun.

[B16] Schaaf B, Rupp J, Muller-Steinhardt M, Kruse J, Boehmke F, Maass M, Zabel P, Dalhoff K (2005). The interleukin-6 -174 promoter polymorphism is associated with extrapulmonary bacterial dissemination in Streptococcus pneumoniae infection. Cytokine.

[B17] Wang J, Robinson JF, Khan HM, Carter DE, McKinney J, Miskie BA, Hegele RA (2004). Optimizing RNA extraction yield from whole blood for microarray gene expression analysis. Clin Biochem.

[B18] Carrol ED, Mankhambo LA, Balmer P, Nkhoma S, Banda DL, Guiver M, Jeffers G, Makwana N, Molyneux EM, Molyneux ME, Smyth RL, Hart CA (2006). Chemokine Responses Are Increased in HIV-Infected Malawian Children With Invasive Pneumococcal Disease. J Acquir Immune Defic Syndr.

[B19] Mouritzen P, Nielsen AT, Pfundheller HM, Choleva Y, Kongsbak L, Moller S (2003). Single nucleotide polymorphism genotyping using locked nucleic acid (LNA). Expert Rev Mol Diagn.

[B20] Vandesompele J, De Preter K, Pattyn F, Poppe B, Van Roy N, De Paepe A, Speleman F (2002). Accurate normalization of real-time quantitative RT-PCR data by geometric averaging of multiple internal control genes. Genome Biol.

